# Antifungal Potential of Secondary Metabolites Derived from *Arcangelisia flava* (L.) Merr.: An Analysis of In Silico Enzymatic Inhibition and In Vitro Efficacy against *Candida* Species

**DOI:** 10.3390/molecules29102373

**Published:** 2024-05-17

**Authors:** Rudi Hendra, Aulia Agustha, Neni Frimayanti, Rizky Abdulah, Hilwan Yuda Teruna

**Affiliations:** 1 Department of Chemistry, Faculty of Mathematics and Natural Sciences, Universitas Riau, Pekanbaru 28291, Indonesia; aulia.agustha6856@grad.unri.ac.id (A.A.); hyteruna@lecturer.unri.ac.id (H.Y.T.); 2 Center of Excellence in Pharmaceutical Care Innovation, Universitas Padjadjaran, Bandung 40600, Indonesia; r.abdulah@unpad.ac.id; 3 Sekolah Tinggi Ilmu Farmasi Riau, Pekanbaru 28293, Indonesia; nenifrimayanti@gmail.com; 4 Department of Pharmacology and Clinical Pharmacy, Faculty of Pharmacy, Universitas Padjadjaran, Jatinangor, Sumedang 45363, Indonesia

**Keywords:** antifungal activity, *Arcangelisia flava*, bioassay-guided fractionation, *Candida* species, molecular docking, pharmacophore modeling, secondary metabolites

## Abstract

Considering the escalating resistance to conventional antifungal medications, it is critical to identify novel compounds that can efficiently counteract this challenge. The purpose of this research was to elucidate the fungicidal properties of secondary metabolites derived from *Arcangelisia flava*, with a specific focus on their efficacy against *Candida* species. This study utilized a combination approach comprising laboratory simulations and experiments to discern and evaluate the biologically active constituents present in the dichloromethane extract of *A. flava*. The in vitro experiments demonstrated that compounds **1** (palmatine) and **2** (fibraurin) exhibited antifungal properties. The compounds exhibited minimum inhibitory concentrations (MICs) ranging from 15.62 to 62.5 µg/mL against *Candida* sp. Moreover, compound **1** demonstrated a minimum fungicidal concentration (MFC) of 62.5 µg/mL against *Candida glabrata* and *C. krusei*. In contrast, compound **2** exhibited an MFC of 125 µg/mL against both *Candida* species. Based on a molecular docking study, it was shown that compounds **1** and **2** have a binding free energy of −6.6377 and −6.7075 kcal/mol, respectively, which indicates a strong affinity and specificity for fungal enzymatic targets. This study utilized pharmacophore modeling and Density Functional Theory (DFT) simulations to better understand the interaction dynamics and structural properties crucial for antifungal activity. The findings underscore the potential of secondary metabolites derived from *A. flava* to act as a foundation for creating novel and highly efficient antifungal treatments, specifically targeting fungal diseases resistant to existing treatment methods. Thus, the results regarding these compounds can provide references for the next stage in antifungal drug design. Further investigation is necessary to thoroughly evaluate these natural substances’ clinical feasibility and safety characteristics, which show great potential as antifungal agents.

## 1. Introduction

Invasive fungal infections are a major logistical challenge, especially for vulnerable populations such as immunocompromised and hospitalized patients. The predominant pathogens responsible for these infections, which cause over 90% of all fatalities associated with fungi, are *Candida*, *Aspergillus*, *Pneumocystis*, and *Cryptococcus*. The escalating resistance these pathogens exhibit toward well-established antifungal medications presents a growing peril to the healthcare sector, underscoring the criticality of developing innovative antifungal agents [1]. The increasing incidence of drug-resistant fungal infections has highlighted the critical need for novel antifungal treatments, especially in healthcare settings [1]. The proposed side effects and the limited efficacy of existing antifungals have further exacerbated the urgency. The remarkable antifungal properties of secondary metabolites derived from *Arcangelisia flava* (L.) Merr. have been postulated in previous research. Using these compounds makes the development of efficacious antifungal treatments against drug-resistant fungal pathogens feasible [2].

Various secondary metabolites derived from *Arcangelisia* species, such as berberine alkaloid and Furanoditerpenes, e.g., fibraurin, have been reported [2]. The investigation of phytochemicals for their potential as antifungal agents has garnered significant interest. Research has documented the considerable efficacy of an ethanol extract derived from the stems of *A. flava* against a wide range of bacterial and fungal strains [3]. The ability of secondary metabolites to obstruct critical biological processes, including protein and cell wall synthesis, is the main factor responsible for these activities [4,5]. In addition, studies have demonstrated that berberine, a compound found in *A. flava*, inhibits these synthesis pathways [6]. For instance, palmatine, a berberine alkaloid, showed antifungal activity against azole-resistant *Candida* strains, with MIC values of 93.46–373.82 μM [7]. An additional instance pertains to fibraurin, which was isolated from *A. flava* and demonstrated antifungal properties against two fungi (*Trametes versicolor* and *Fomitopsis palustris*) at a concentration of 1000 µg/mL [8].

Notwithstanding these observations, there continues to be significant knowledge regarding the precise secondary metabolites that underlie the antifungal properties of *A. flava*, specifically with *Candida* species. Prior research has not exhaustively investigated the process of isolating and characterizing these bioactive compounds, nor has it specified the precise mechanisms by which they exert their effects on fungal pathogens. Antifungal activity against Candida species has been observed in the dichloromethane extract derived from the root of *A. flava*, with minimum inhibitory concentrations (MICs) ranging from 250 to 500 µg/mL [9]. This finding indicates the potential of the extract as a source of antifungal agents.

This study aimed to isolate and characterize the secondary metabolites found in *A. flava* dichloromethane extract, as well as their antifungal properties. In vitro assessment was conducted to determine the antifungal potency of the isolated compounds against a range of *Candida* species. Furthermore, an evaluation of these compounds’ potential inhibitory impacts on crucial fungal enzymes, including Lanosterol 14-alpha demethylase (3LD6), was performed using in silico analyses. The overarching objective of this research was to contribute to developing targeted antifungal therapies that effectively combat drug-resistant fungal pathogens. The investigation combined chromatographic and spectroscopic methodologies to extract and define the bioactive compounds. Using established in vitro assays, the antifungal activities of the isolated compounds were evaluated, and their mechanisms of action were analyzed via in silico docking studies targeting particular fungal enzymes. The primary objective of this all-encompassing strategy was to discern novel prospective antifungal agents and gain insight into their mechanism of action, which is a critical component in advancing targeted and efficacious treatments.

## 2. Results

### 2.1. Extraction and Isolation

Previous research found that a dichloromethane extract obtained from the roots of *A. flava* demonstrated promising antifungal properties against three different *Candida* species. Based on this preliminary identification, the current study conducted another round of extract purification using vacuum liquid chromatography (VLC). At the same time, the procedure was closely monitored using thin-layer chromatography (TLC), resulting in the formation of nine discrete fractions. The antifungal efficacy of these fractions was then evaluated. Notably, fraction six (F6) exhibited the most significant antifungal activity, as detailed in Table 1.

An additional examination was conducted on fraction F6, which exhibited the appearance of a yellow–orange solid. A purification procedure was implemented on this fraction utilizing the recrystallization technique. By following this systematic procedure, two compounds were successfully isolated: compound **1**, which was obtained at a volume of 910 mg, and compound **2**, which was obtained at a lesser yield of 42 mg. The NMR spectroscopy data for compounds **1** and **2** are presented in the Supplementary Materials (Figures S1 and S2) and compared with previous publications [8,10].

### 2.2. Antifungal Activities

The current investigation evaluated the antifungal effectiveness of various fractions derived from the dichloromethane extract and compounds **1** and **2**. *C. krusei* ATCC 14243, *C. albicans* ATCC 10231, and *C. glabrata* ATCC 15126 constituted the targets of this assay. The findings suggest that most fractions (F1–F9) exhibited minimum inhibitory concentration (MIC) values ranging from moderate to high when tested against three *Candida* species. However, F6 and compounds **1** and **2** displayed the most potent antifungal activity. Additionally, fraction F5 exhibited uniform activity among all species. As suggested by the minimum fungicidal concentration (MFC) data, several fractions exhibited negligible fungicidal activity, specifically against *C. albicans* and *C. glabrata*. On the contrary, F6 and compounds **1** and **2**, with ketoconazole in particular, demonstrated reduced MFCs, suggesting more potent fungicidal characteristics. As a control, ketoconazole consistently showed the lowest MIC and MFC values (Table 1).

### 2.3. Molecular Docking

Lanosterol 14alpha-demethylase (PDB ID 3LD6) was used as a receptor for the molecular docking. The enzyme lanosterol 14-alpha demethylase (PDB ID 3LD6) is a key target in antifungal research due to its role in ergosterol biosynthesis, a crucial component of fungal cell membranes [11]. In *Candida* species, ergosterol is essential for cell membrane integrity and fluidity, and its biosynthesis is regulated at multiple levels [12]. The binding pocket of lanosterol 14-alpha demethylase has been identified as a key site for antifungal drug design [13]. The docking results for these ligands with ketoconazole, which was employed as a positive control against lanosterol 14alpha-demethylase (PDB ID 3LD6), are presented in Table 2. Molecular docking is a widely used silico technique in drug discovery with two main functions: firstly, it predicts the behavior of a novel chemical; secondly, it observes ligands that can serve as potent inhibitors by examining the interactions between each ligand and the target protein. This method allows researchers to obtain valuable information about ligands’ binding strength and selectivity, which can contribute to the development of and improvement to new therapeutic drugs. The optimal docking pose was determined in this study by considering specific parameters, including the lowest binding free energy and a root mean square deviation of less than 2 [14,15].

### 2.4. Pharmacophore

**Figure 1:**
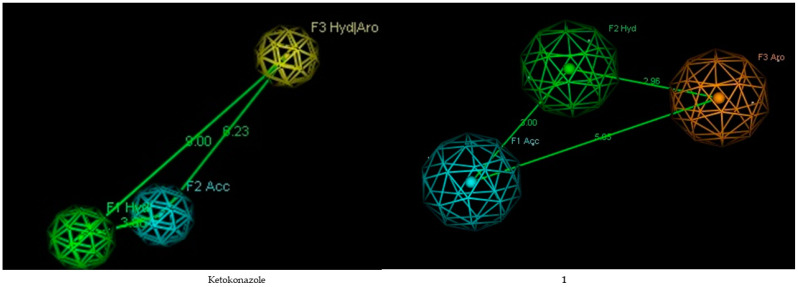
The ideal pharmacophore hypothesis. The yellow sphere represents an aromatic ring, the green sphere represents a hydrophobic group, and the blue sphere represents a hydrogen acceptor.

A pharmacophore is a collection of steric and electronic characteristics crucial for enabling interactions between a supramolecular entity and a biological target. Our investigation successfully found three crucial pharmacophore characteristics, as depicted in Figure 1. The features encompass hydrophobic areas (shown in the color green), hydrogen bond donors (represented by the color yellow), and aromatic rings (represented by the color orange). These properties are essential for assessing the activity of compounds, indicating their importance in the context of active chemicals.

### 2.5. Density Functional Theory

DFT calculations were conducted to optimize the gas-phase structures of compounds **1** and **2**. These two compounds were selected because of their lowest binding free energy, as demonstrated by the docking findings. The objective of this optimization was to enhance the molecular configurations and gain an understanding of their energetically favorable conformations without considering solvent interactions. The DFT calculation is displayed in Table 3.

## 3. Discussion

*Arcangelisia flava* (L.) Merr., a member of the Menispermaceae family, exhibits a broad distribution spanning from China to New Guinea, thereby establishing its quasi-native status across Southeast Asia. The distinctive feature of this Borneo-native species is its brilliant yellow wood. The plant, known locally as k‘ikoneng’ in West Java and k‘ayo kuning’ in Palembang, Indonesia, has historically been used to remedy many ailments. It treats thrush, fever, diarrhea, hepatitis, worm infections, and gastrointestinal disorders. The Dayak Ot Danum tribe residing in Tumbang Payang, Kalimantan, utilizes the roots and stems of *A. flava* as a remedy for jaundice/hepatitis and other ailments affecting the liver [16].

Due to its exceptional performance, the dichloromethane extract of *A. flava* was selected as the focus of this study. The extract was purified using the bioassay-guided fractionation method, a highly regarded and widely recognized technique in drug discovery research. The efficacy of this method is due to its strategic approach of establishing a direct correlation between the analyzed extract and the target compounds. The bioassay-guided fractionation technique utilizes a rigorous fractionation process and precise biological activity evaluations. By employing this methodical strategy, every fraction undergoes assessment regarding its potential therapeutic attributes, thus simplifying, identifying, and isolating compounds with the intended biological effects. By combining the fractionation and bioassay procedures, scientists can enhance the accuracy of and determine the active components present in the extract, thereby enabling a more focused and fruitful investigation of possible pharmacological agents [17,18].

Compound **1** was comprehensively characterized by UV–Vis, FT-IR, and NMR spectroscopy techniques. According to the UV–Vis spectroscopic analysis, the maximum absorption was observed at wavelengths of 266 nm, 350 nm, and 426 nm, which indicates the existence of conjugated double bonds. By acting as chromophores, these bonds facilitate the molecule’s efficient absorption of ultraviolet light.

Additional knowledge was acquired via FT-IR spectroscopy, which unveiled a discernible absorption at 3322 cm^−1^, suggesting that the amino N-H group is frequently encountered in alkaloids. A further observation of absorption at 1277 cm^−1^ supported the presence of a C-N group. Additionally, absorptions at 3069 cm^−1^ and 2948 cm^−1^, which correspond to aromatic and aliphatic C-H functional groups, respectively, were observed in the spectrum. Significant absorption confirmed the presence of a C=C alkene functional group at 1633 cm^−1^. This was further corroborated by absorption at 1523 cm^−1^, which indicated the existence of an aromatic C=C bond. A final indicator of a C-O functional group was the absorption peak at 1112 cm^−1^.

**Figure 2:**
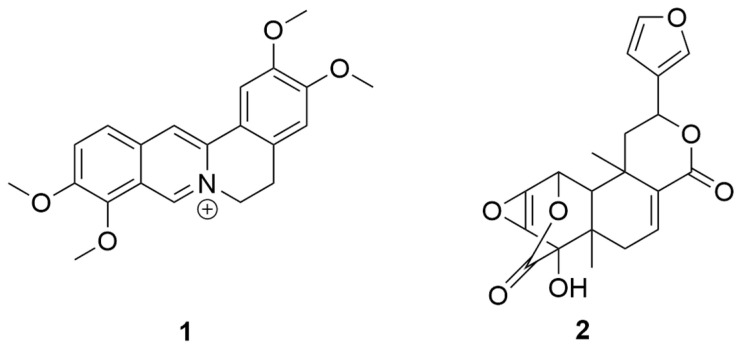
Structures of compounds. Compound **1**: palmatine, Compound **2**: fibraurin.

Compound **1**’s ^1^H NMR spectrum revealed the presence of six aromatic protons at δ 9.91, 9.09, 8.21, 8.04, 7.72, and 7.09. Furthermore, four methoxyl groups were detected at δ 4.10, 4.07, 3.94, and 3.87, suggesting that methylation has occurred within the molecular structure. Additionally, the spectrum revealed two sets of methylene protons at δ 4.95 and 3.22, which provided additional insights into the compound’s structural characteristics. The ^13^C NMR spectrum provided additional information by exposing twenty-one unique carbon atoms. The spectroscopic properties of compound **1** exhibited a striking similarity to the protoberberine skeleton, as determined by a comprehensive examination and comparison with the existing literature, with particular reference to Gao et al. (2008) [10]. Compound **1**, illustrated in Figure 2, was identified as palmatine by combining this alignment and the specific NMR data (see Supplementary Materials).

The FT-IR spectroscopy revealed significant absorptions in compound **2**: C-H bonds at 3136 cm^−1^, -CH_3_ groups at 2989 and 2968 cm^−1^, a C=C bond at 2360 cm^−1^, and C=O groups at 1768 and 1693 cm^−1^. The ^1^H NMR spectrum revealed signals at 7.77, 7.69, and 6.65, which were attributed to an α,β-substituted furan moiety; it also showed two sets of geminal protons at 2.32, 1.72, 2.29, and 1.98, and two methyl group singlets at 1.17 and 1.06. A hydroxyl group proton appeared at 6.50, as did epoxide protons at 3.86 and 3.68. Moreover, in the ^1^H NMR spectrum, four methine resonances were detected at 7.25, 5.67, 5.10, and 1.77. The ^13^C NMR spectrum revealed 20 carbons, two of which were carbonyl carbons at 171.5 and 163.2. These NMR data, consistent with furanoditerpenoids and supported by the studies of Su et al. (2008) and Suzuki et al. (2011), confirmed the identity of compound **2** as fibraurin (Figure S2, see Supplementary Materials) [8,19].

The present research investigated the minimum inhibitory concentration (MIC) and minimum fungicidal concentration (MFC) of two isolated compounds (**1** and **2**) and nine fractions (F1–F9) in addition to the standard antifungal ketoconazole. The investigation was conducted against three strains of *Candida*: *C. albicans*, *C. glabrata*, and *C. krusei*. The antifungal effects of the majority of fractions were moderate, with MICs primarily observed at 250 µg/mL. However, sub-fraction F6 demonstrated superior efficacy against all the species, exhibiting an MIC of 125 µg/mL. Significantly, sub-fraction F6 showed the highest potency, specifically against *C. krusei* and *C. albicans*, as evidenced by its MIC of 62.5 µg/mL. With a minimum inhibitory concentration (MIC) of 15.62 µg/mL, compound **1** exhibited significant efficacy against *C. glabrata* among the isolated compounds. Similarly, compound **2** demonstrated consistent activity against all the species, with an MIC of 31.25 µg/mL. As a control, ketoconazole demonstrated its potent antifungal properties by achieving the lowest MIC of 7.81 µg/mL across all the tested species.

With MFCs exceeding 250 µg/mL, most of the fractions exhibited limited fungicidal activity against *C. albicans* and *C. glabrata*. However, with MFCs around 250 µg/mL, they demonstrated marginally improved efficacy against *C. krusei*. Sub-fraction F6 remarkably retained its superior activity with an MFC of 125 µg/mL across all the species. With MFCs of 125 and 62.5 µg/mL, compound **1** demonstrated potent fungicidal activity against *C. albicans* and *C. glabrata*, respectively. In contrast, compound **2** maintained a consistent MFC of 62.5 µg/mL against *C. albicans* and higher values for the remaining compounds. Compound **1**, a naturally occurring isoquinoline alkaloid, has been reported to be antibacterial, specifically targeting Gram-positive bacteria [20]. Nevertheless, its antifungal properties are not very strong. Compound **1** showed very minimal effectiveness against *C. albicans*, with a minimum inhibitory concentration (MIC) value of over 1000 µg/mL [21].

Previous studies have documented the presence of compound **1**, which has been identified as palmatine, in *A. flava* [22] and *A. gusanlung* [23], in addition to several families, including Annonaceae, Berberidaceae, Papaveraceae, and Ranunculaceae [24]. The compound previously showed antifungal activity against azole-resistant *Candida* strains, with MIC values of 93.46–373.82 μM [7]. Moreover, compound **1** has demonstrated promising efficacy as a West Nile virus inhibitor and a preventive drug against liver injury [25,26]. Furthermore, the compound has been extensively studied for its pharmacological properties, including its potential to treat jaundice, liver-related diseases, hypertension, inflammation, and dysentery [24]. It also protects against metabolic syndrome and related complications, such as cardiovascular diseases, osteoporosis, and osteoarthritis [7]. Furthermore, according to the research conducted by Ali and Ali (2014), palmatine has a notable impact on human skin epithelial carcinoma cells by causing oxidative stress and DNA damage. The data demonstrate a positive correlation between concentration and time regarding cytotoxicity and an increase in intracellular reactive oxygen species and enzyme activities associated with oxidative stress. Consequently, this leads to substantial DNA damage. The evident potential of palmatine as an anticancer agent suggests its usefulness in cancer therapy. Nevertheless, the genotoxicity of the substance also warrants caution due to its potential to induce secondary carcinogenic processes. This highlights the inherent duality of natural compounds such as palmatine, emphasizing the importance of thoroughly assessing their impact on cells in the context of cancer therapy [27,28].

Compound **2** has also been identified in *Fibraurea tinctoria* [19] and *A. flava* [8], among other species. Fibraurin, which was isolated from *A. flava*, demonstrated antifungal properties against two fungi (*Trametes versicolor* and *Fomitopsis palustris*) at a concentration of 1000 µg/mL [8]. However, there are no documented investigations into its antifungal properties, specifically against *Candida* species. Furthermore, the compound has been identified as a potential treatment for Alzheimer’s disease due to its ability to act on anti-AD targets [29]. This compound is part of a group of bioactive compounds with therapeutic activities, including antioxidant, anti-inflammatory, and cardiovascular protective effects [30].

The present study examined the affinity of these two isolated compounds as possible inhibitors of antifungal activity. An in silico investigation was performed using the MOE 2022.0901 software package to investigate the chemical and protein interactions. The docking results were compared with those of ketoconazole, which was used as a positive control drug. The docking approach was used to clarify the interactions between the chemicals and the protein’s active site.

Table 2 displays the energy values based on the molecular docking study. The results show that all the compounds had lower energies than the positive control molecule (ketoconazole). Significantly, compounds **1** and **2** exhibited the most minimal binding free energy. Molecule **1** demonstrated a binding free energy of −6.637 kcal/mol, whereas the positive control molecule had a binding free energy of −1.6989 kcal/mol. Compound **2** exhibited a binding free energy of −6.7075 kcal/mol. Smaller binding energy values indicate a higher probability of ligand–receptor binding.

These data indicate a significant likelihood of molecular interactions between proteins and these chemicals. In addition, due to their higher binding free energy than the positive control compound, compounds **1** and **2** show potential as viable candidates for future antifungal treatments [31,32].

**Figure 3:**
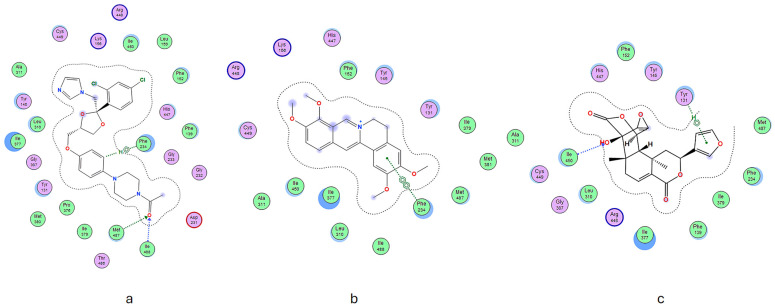
The spatial arrangement of (**a**) ketoconazole, (**b**) compound **1**, and (**c**) compound **2**.

Compound **1** exhibits specific amino acid residues, namely, Phe234 and Arg448, that resemble the positive control compound. These residues are situated in the active site and engage in hydrogen bonding. Compound **2** formed hydrogen bonds with amino acid residues, such as Tyr131, while compound **1** distinctly interacted with Ile450. These different interactions may contribute to the inhibitory effects of these substances. Significantly, these separate chemicals participate in hydrogen bonding, as evidenced by our docking results (see Figure 3).

In addition, hydrophobic and van der Waals interactions significantly enhanced these chemicals’ ability to dock into protein-binding pockets. The results emphasize the complex nature of how ligands and proteins interact and demonstrate the potential of these chemicals to be effective inhibitors through numerous modes of interaction.

The pharmacophore study of ketoconazole, used as a positive control, revealed the presence of crucial properties necessary for ligand–receptor interactions. These features include a hydrogen bond acceptor group (F2:Acc), an aromatic ring (F3:Aro), and a hydrophobic group (F1:Hyd). The distance between the hydrogen bond acceptor group (F2:Acc) and the hydrophobic group (F1:Hyd) is 3.60 Å. The distances between the hydrogen bond acceptor group (F2:Acc) and the aromatic group (F3:Aro), and between the hydrophobic group (F1:Hyd) and the aromatic group (F3:Aro), are 6.23 Å and 9.00 Å, respectively.

Compounds **1** and **2** share similar properties with the positive control molecule, including a hydrogen bond acceptor group (F6:Acc), an aromatic ring (F5:Aro), and a hydrophobic group (F4:Hyd). The hydrogen bond acceptor group (F6:Acc) is 3.00 Å away from the hydrophobic group (F4:Hyd). The distances between the hydrogen bond acceptor group (F6:Acc) and the aromatic group (F5:Aro), and between the hydrophobic group (F4:Hyd) and the aromatic group (F5:Aro), are 5.05 Å and 2.96 Å, respectively. Adding a hydrophobic component to a ligand significantly affects the molecule’s effectiveness. A reduced distance indicates superior results [33].

The DFT and B3LYP techniques [34,35] were used to optimize the molecular structure of compounds **1** and **2** in the gas phase. This was conducted using the 6–31 G basis set, which was implemented using the Gaussian 5.0 software package. The chemical stability of the two compounds was evaluated by employing the highest occupied molecular orbital (HOMO) and lowest unoccupied molecular orbital (LUMO) [8]. The energy gap and chemical reactivity descriptors were calculated using the DFT/B3LYP/6-31G method. The findings of the DFT and B3LYP analysis are displayed in Table 3.

According to the present study, compounds **1** and **2** have energy gaps of 0.097 and 0.012 eV, respectively. A negligible disparity in energy levels between the lowest unoccupied molecular orbital (LUMO) and the highest occupied molecular orbital (HOMO) signifies that the compound is chemically unstable, possesses a low degree of stability, and is susceptible to perturbation; thus, this characteristic enhances its biological efficacy. The EHOMO denotes the electron-donating capability of the object, whereas the ELUMO denotes the electron-accepting capability. Further support for these conclusions was provided by the results obtained from molecular coupling experiments.

## 4. Materials and Methods

### 4.1. Plant Collection

The roots of *A. flava* were sourced from Ranah Sungkai Village, XIII Koto Kampar District, Kampar Regency, at coordinates 0.390662541132676° N, 100.71270015089992° E. Prof. Fitmawati of the Department of Biology, Universitas Riau, identified this specimen of *A. flava*. A voucher specimen bearing the number 508/UN19.5.1.1.3-4/EP/2022 was deposited at the Laboratory of Botany within the Department of Biology at Universitas Riau, Indonesia.

### 4.2. Extraction and Isolation

The chopped roots of *A. flava* were subsequently extracted using the maceration method. A total of 500 g of the roots was placed into a bottle, followed by adding 1 L of methanol solvent to ensure all samples were fully submerged. The bottle was then shaken for a brief period. The samples underwent maceration for 3 × 24 h, with shaking occurring twice daily. The macerated samples were then filtered using cheesecloth and cotton. The maceration process was repeated by adding methanol to the filtered samples and shaking the samples until a colorless extract was produced.

The extract obtained from the filtration was then concentrated using a rotary evaporator until all the methanol solvent had evaporated from the sample. Subsequently, the extract was separated using the liquid–liquid extraction method with *n*-hexane, dichloromethane, and ethyl acetate as the solvents, resulting in n-hexane, dichloromethane, ethyl acetate, and water fractions.

Antifungal testing was performed on each fraction, and the dichloromethane fraction exhibited the most promising results. Afterward, the dichloromethane fraction was purified using vacuum liquid chromatography (VLC). Approximately 35 g of the fraction was separated in silica using a solvent system of *n*-hexane–ethyl acetate–methanol. The separation process was monitored using thin-layer chromatography (TLC), resulting in nine sub-fractions. Antifungal assays were conducted on each sub-fraction, revealing that sub-fraction F6 displayed significant antifungal activity. Fraction six was subsequently purified through recrystallization using dichloromethane. The solution and the precipitation were separated through decantation, yielding compound **2** in the solution and compound **1** as a yellow solid.

### 4.3. In Silico Studies

#### 4.3.1. Protein Preparation

The protein was downloaded from the Protein Data Bank (RSCB PDB) website under the PDB ID 3LD6 (lanosterol 14alpha-demethylase). The protein preparation process commenced by eliminating the initial ligand from the protein structure and adding hydrogen atoms. Subsequently, the alpha carbon atom and backbone atoms underwent depreciation, and finally, the entire protein molecule underwent minimization. This minimization procedure utilized the CHARMM27 force field with a distance parameter of 0.01.

#### 4.3.2. Ligand Preparation

Two ligands and ketoconazole (positive control) were drawn using ChemDraw 15.0 software. Subsequently, all the ligand structures were imported into Discovery Studio Visualizer and then transferred to MOE 2022.0901 software. In the MOE software package (Chemical Computing Group), the ligand files were integrated into the mdb database. This enabled the ligands to be utilized for conducting docking simulations.

#### 4.3.3. Molecular Docking

Before conducting molecular docking, it is crucial to determine the protein’s binding site. A site finder tool was utilized to identify active sites within the protein. Sites 2 and 8 were designated as the target sites for the docking process. In the docking setup, the target site was set as a dummy atom in the docking menu, and the ligand structures were chosen from the MDB file. Refinement was rigid, with posture settings at 50 and 10 and placement set as a triangle. Subsequently, the docking process was initiated.

#### 4.3.4. Generation of Pharmacophore

The pharmacophore query editor tool was employed to establish the pharmacophore characteristics of the synthesized compounds. This tool facilitated the identification of key pharmacophore features and the formulation of hypotheses regarding their alignments. Specifically, in this study, the pharmacophore alignment was based on three primary features: hydrophobicity, hydrogen bond donation, and aromatic ring presence.

#### 4.3.5. Density Functional Theory

The DFT calculations utilized the Becke three-parameter hybrid functional (B3LYP), the 6-31G basis set, and the Lee–Yang–Parr correlation functional (B3LYP) methods, executed within the Gauss View 5 software platform. The investigation involved optimizing geometries, analyzing frequencies, and producing molecular electrostatic potential (MEP) maps for individual molecules. Before optimizing the geometries, a conformational search was conducted to investigate potential conformations. Furthermore, frequency analysis was conducted for each structure to validate that the optimized geometries represented true minima.

### 4.4. In Vitro Antifungal Activity

#### 4.4.1. Preparation of Media

Sabouraud Dextrose Agar (SDA) and Sabouraud Dextrose Broth (SDB) were used to grow fungus. SDA was made by dissolving 65 g in 1 L of distilled water, and SDB was made by dissolving 30 g in the same volume. Both media were sterilized via autoclaving after being boiled on a hotplate.

#### 4.4.2. Cultivating Fungi

Cultures of *Candida albicans* ATCC 10231, *C. glabrata* ATCC 15126, and *C. krusei* ATCC 14243 were grown on SDA slant agar by using a cross-streaking method. The cultures were incubated at around 37 °C for 1–2 days to encourage their revival and expansion.

#### 4.4.3. Minimum Inhibitory Concentration (MIC)

The minimum inhibitory concentration (MIC) was determined using a 96-well microplate and the microdilution technique. Cultures of *C. albicans*, *C. glabrata*, and *C. krusei* were adjusted to an optical density of approximately 0.02 at 530 nm. Serial dilutions of the samples were made at a two-fold dilution. Each well in the microplate was filled with 50 μL of the medium, 10 μL of resazurin indicator, and 10 μL of the diluted fungal culture. Control wells were also prepared, including positive (ketoconazole) and negative controls. The microplate was incubated at approximately 37 °C for 24 h. The minimum inhibitory concentration (MIC) was determined by visually examining color changes in the wells and comparing them to the negative control [36].

#### 4.4.4. Minimum Fungicidal Concentration (MFC)

The MFC was identified using SDA plates. Each plate was filled with 25 mL of SDA and left to solidify. Next, 10 μL of each solution at their minimum inhibitory concentration (MIC) was spread across the SDA surface. The plates with the inoculum were placed in an incubator at 37 °C for 24 h. MFC determination was visually accomplished by assessing the lack of fungal colonies and the clarity of the SDA medium.

## 5. Conclusions

This study investigated the effectiveness of secondary metabolites obtained from *A. flava*, specifically palmatine (**1**) and fibraurin (**2**), in combating *Candida* species. The research combined laboratory experiments with computer-based studies. The results showed that both compounds exhibited antifungal properties, as indicated by their minimum inhibitory concentrations (MICs). Palmatine (**1**) demonstrated the highest efficacy, exhibiting the lowest minimum inhibitory concentration (MIC) value against *C. glabrata*. In addition, Density Functional Theory (DFT) simulations were employed to examine the electronic characteristics of these compounds. The simulations revealed that the compounds have low energy gap values, indicating a high level of reactivity. This reactivity could potentially contribute to their antifungal activities. The findings highlight the importance of secondary metabolites from *A. flava* as potential candidates for developing new antifungal drugs, which is particularly significant due to the increasing problem of antifungal resistance. The precise interactions between these metabolites and fungal enzymes, elucidated through molecular docking, offer valuable insights into their mode of action, thereby establishing a fundamental understanding that can guide future drug development. Future research should prioritize comprehensive evaluations to validate the safety and effectiveness of these compounds. Additionally, investigations should examine the potential of combining these compounds with other therapies to enhance their ability to fight fungal infections and reduce the development of resistance. Furthermore, it is essential to conduct additional research on the pharmacokinetics of these compounds and determine the most effective dosage to advance their potential as medically beneficial medications. The positive findings from this study support a further exploration of the extensive pharmacological capabilities of natural products in addressing urgent requirements in antifungal treatment strategies.

## Figures and Tables

**Table 1:** Antifungal activity of different fractions and compounds **1** and **2**.

Sample	MIC (µg/mL)			MFC (µg/mL)		
	*C. albicans*	*C. glabrata*	*C. krusei*	*C. albicans*	*C. glabrata*	*C. krusei*
F1	250	>250	125	>250	>250	250
F2	250	250	>250	>250	>250	250
F3	250	>250	125	>250	>250	250
F4	250	>250	125	>250	>250	250
F5	125	125	125	>250	>250	250
F6	62.5	125	62.5	125	125	125
F7	250	125	125	>250	250	250
F8	250	250	125	>250	>250	250
F9	250	125	125	>250	250	250
**1**	62.5	15.62	31.25	125	62.5	62.5
**2**	31.25	31.25	31.25	62.5	125	125
Ketoconazole	7.81	7.81	7.81	7.81	7.81	7.81

**Table 2:** Docking results.

Compound	Binding Free Energy (kcal/mol)	RMSD	H Bond	Hydrophobic Interaction	Van der Waals Interaction	Other Interactions	Binding Factor
**Ketoconazole**	−1.6989	1.1078	Phe234, Met487, Ile488	Lys156, Arg448	Asp231	Ala311, Tyr145, Leu310, Ile377, Gly307, Tyr131, Met380, Pro376, Ile379, Thr486, Gly232, Gly233, Phe139, His447, Phe152, Leu159, Ile450, Cys449	**24**
**1**	−6.6377	1.1398	Phe234	Lys156, Arg448	-	Cys449, Ala311, Ile450, Ile377, Leu310, Ile488, Met487, Met381, Ile379, Tyr131, Tyr145, Phe152, His447	15
**2**	−6.7075	0.6158	Tyr131, Ile450	Arg448	-	Ala311, Cys449, Gly307, Leu310, Ile377, Phe139, Ile379, Phe234, Met487, Tyr145, Phe152, His447	15

**Table 3:** DFT results.

Compound	Energy	Electronic Structure		Energy Gap
		HOMO	LUMO	
**1**	−1597.23	−0.189	−0.092	0.097
**2**	−1803.12	−0.210	−0.198	0.012

## Data Availability

The data can be accessed either through a repository or online through the data retention policies provided by the funding entities.

## Supplementary Materials

The following supporting information can be downloaded at https://www.mdpi.com/article/10.3390/molecules29102373/s1; the online version contains supplementary material featuring NMR spectral data of compounds **1**–**2**, including 2D NMR spectral data for these compounds.
