# Supplementary material for: Antifungal Potential of Secondary Metabolites Derived from Arcangelisia flava (L.) Merr.: An Analysis of In Silico Enzymatic Inhibition and In Vitro Efficacy against Candida Species
Source: Molecules. 2024 May 17;29(10):2373. doi: 10.3390/molecules29102373 (PMC11123860; doi:10.3390/molecules29102373)

Supporting Information For

Antifungal Potential of Secondary Metabolites Derived from *Arcangelisia flava* (L.) Merr.: An Analysis of Enzymatic Inhibition *in Silico* and *In Vitro* Efficacy Against *Candida* Species

Rudi Hendra [1,2], Aulia Agustha [1], Neni Frimayanti[3], Rizky Abdulah[2,4], and Hilwan Yuda Teruna[1]

1 Department of Chemistry, Faculty of Mathematics and Natural Sciences, Universitas Riau, Pekanbaru, Indonesia
2 Center of Excellence in Pharmaceutical Care Innovation, Universitas Padjadjaran, Bandung, Indonesia
3 Sekolah Tinggi Ilmu Farmasi Riau, Pekanbaru, Indonesia
4 Department of Pharmacology and Clinical Pharmacy, Faculty of Pharmacy, Universitas Padjadjaran, Jatinangor, Indonesia

* Correspondence: rudi.hendra@lecturer.unri.ac.id

Table of Contents

Figure S1. 1H-NMR spectrum of compound 1 (DMSO-d6) 2
Figure S2. ^{13}C-NMR spectrum of compound **1** (DMSO-d6) 3
Figure S3. HSQC spectrum of compound **1** (DMSO-d6) 4
Figure S4. HMBC spectrum of compound **1** (DMSO-d6) 5
Figure S5. COSY spectrum of compound **1** (DMSO-d6) 6
Figure S6. ^{1}H-NMR spectrum of compound **2** (DMSO-d6) 7
Figure S7. ^{13}C-NMR spectrum of compound **2** (DMSO-d6) 8
Figure S8. HSQC spectrum of compound **2** (DMSO-d6) 9
Figure S9. HMBC spectrum of compound **2** (DMSO-d6) 10
Figure S10. COSY spectrum of compound **2** (DMSO-d6) 11

Figure S1. 1H-NMR spectrum of compound 1 (DMSO-d6)

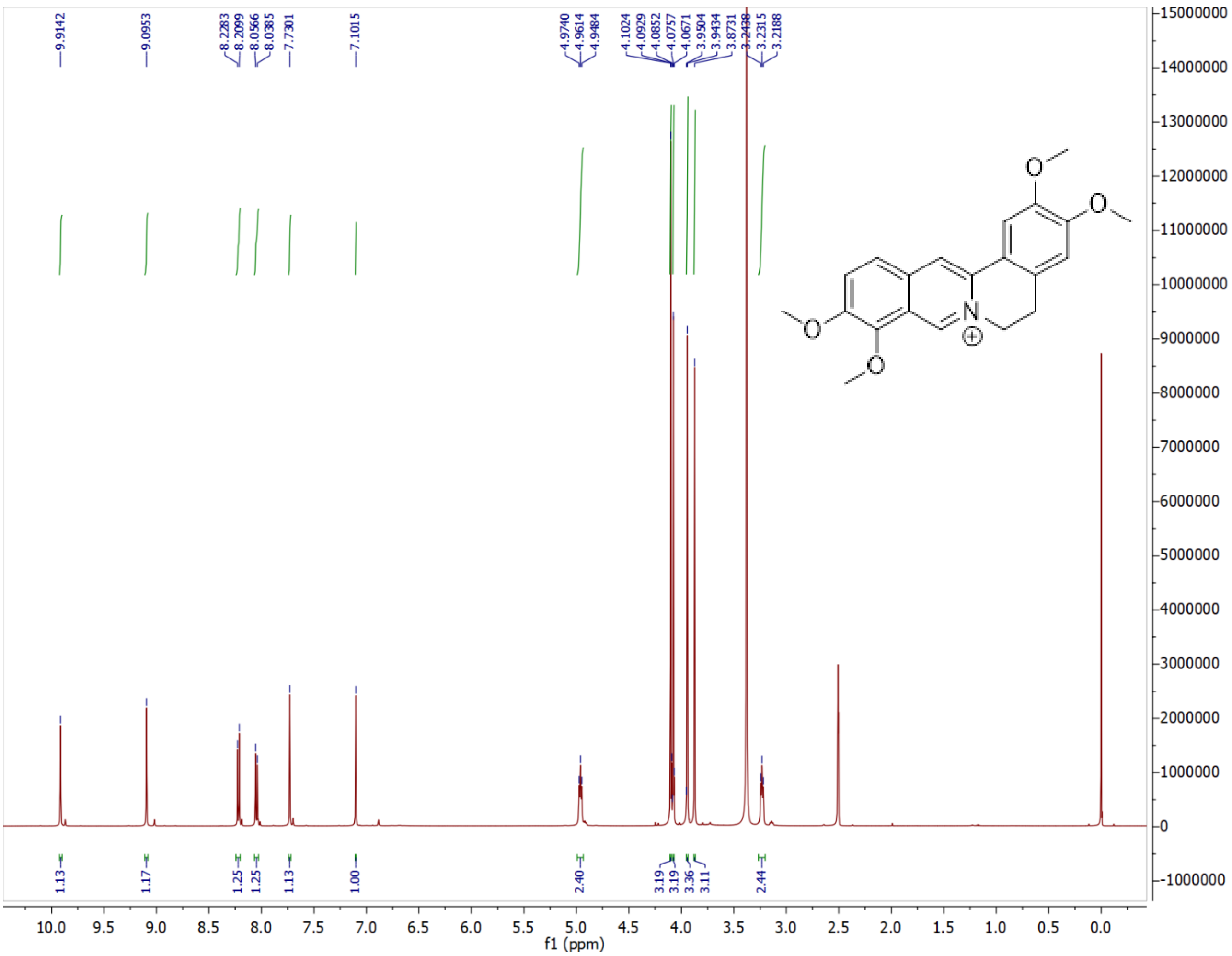

Figure S2. ^{13}C-NMR spectrum of compound **1** (DMSO-d6)

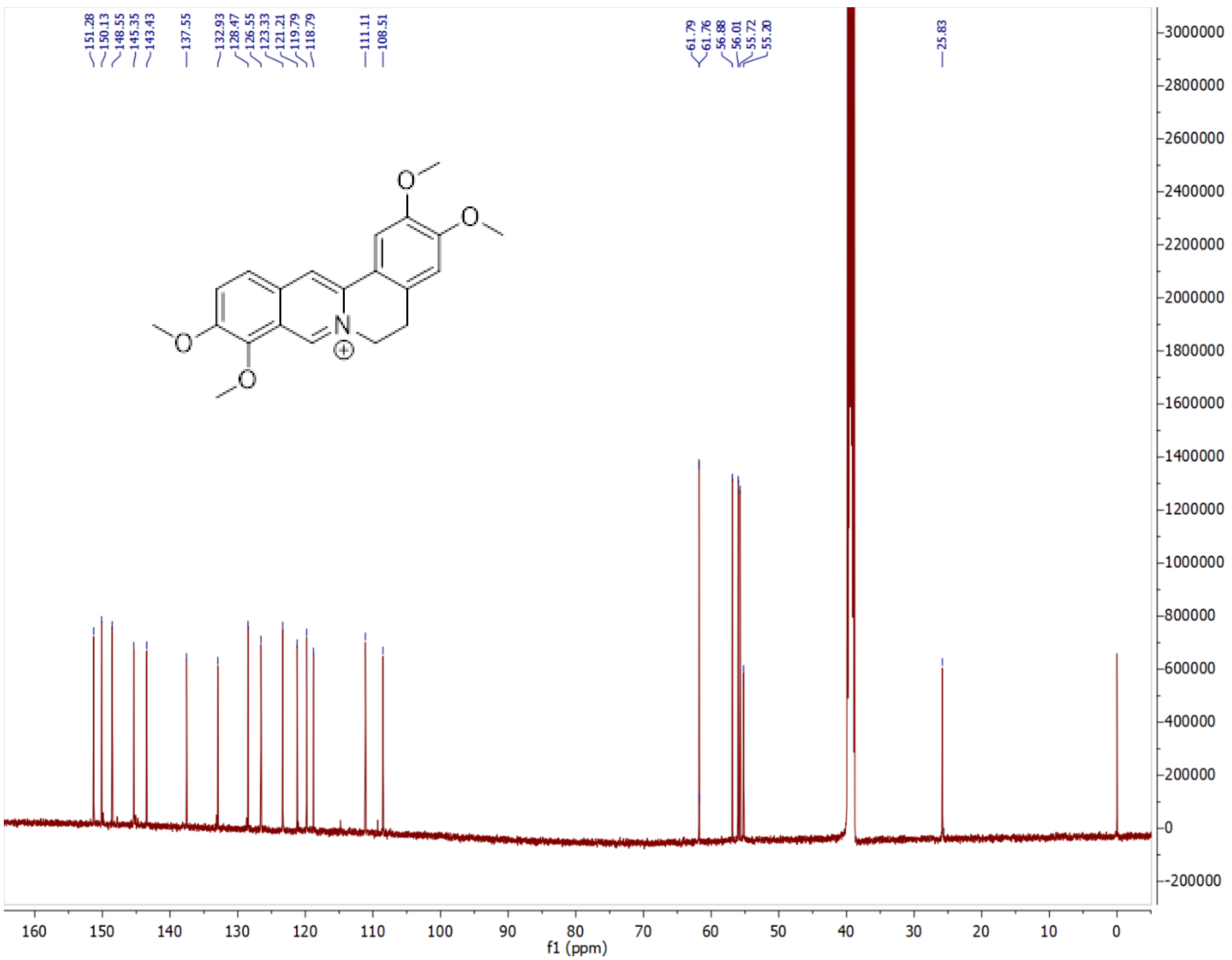

Figure S3. HSQC spectrum of compound **1** (DMSO-d6)

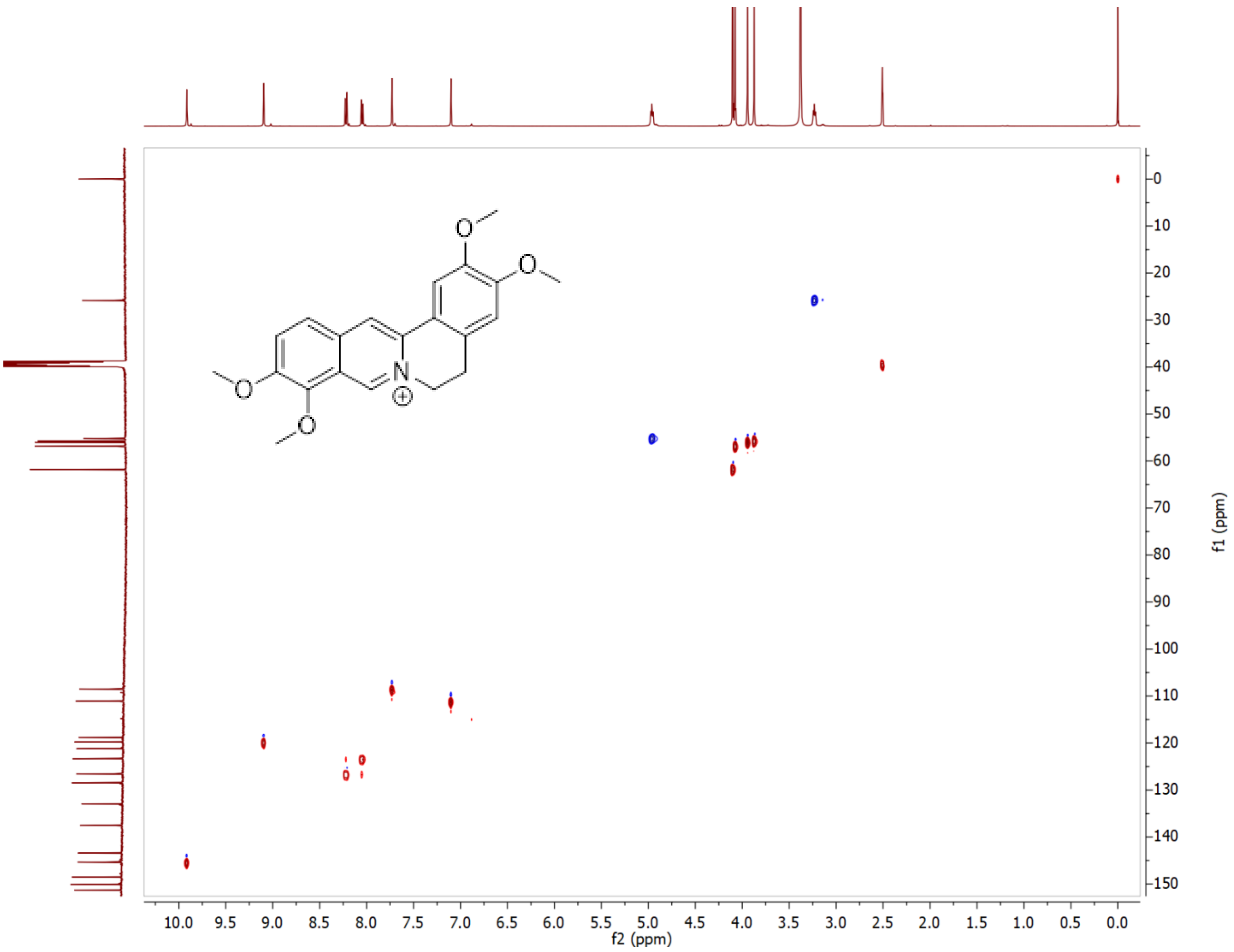

Figure S4. HMBC spectrum of compound **1** (DMSO-d6)

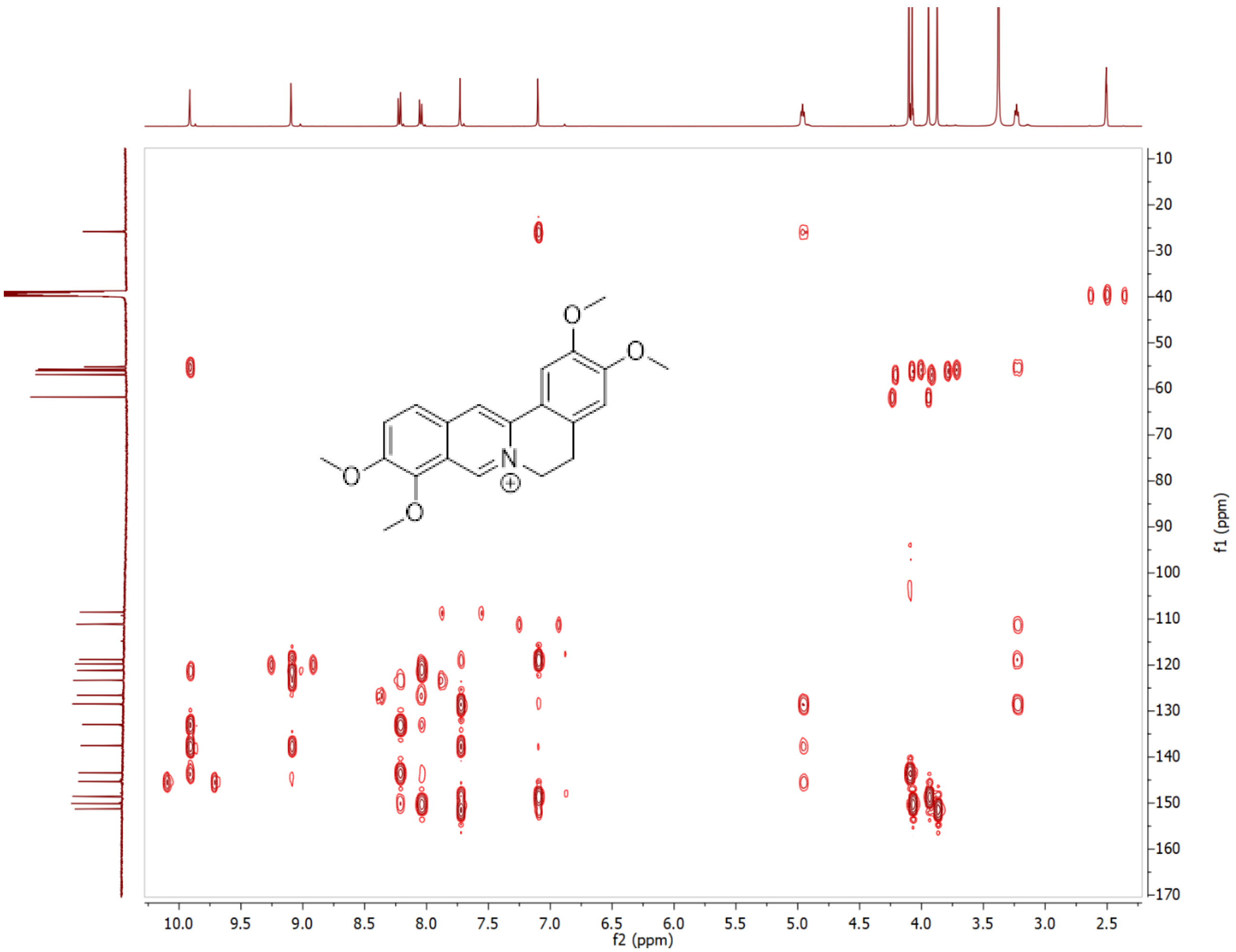

Figure S5. COSY spectrum of compound **1** (DMSO-d6)

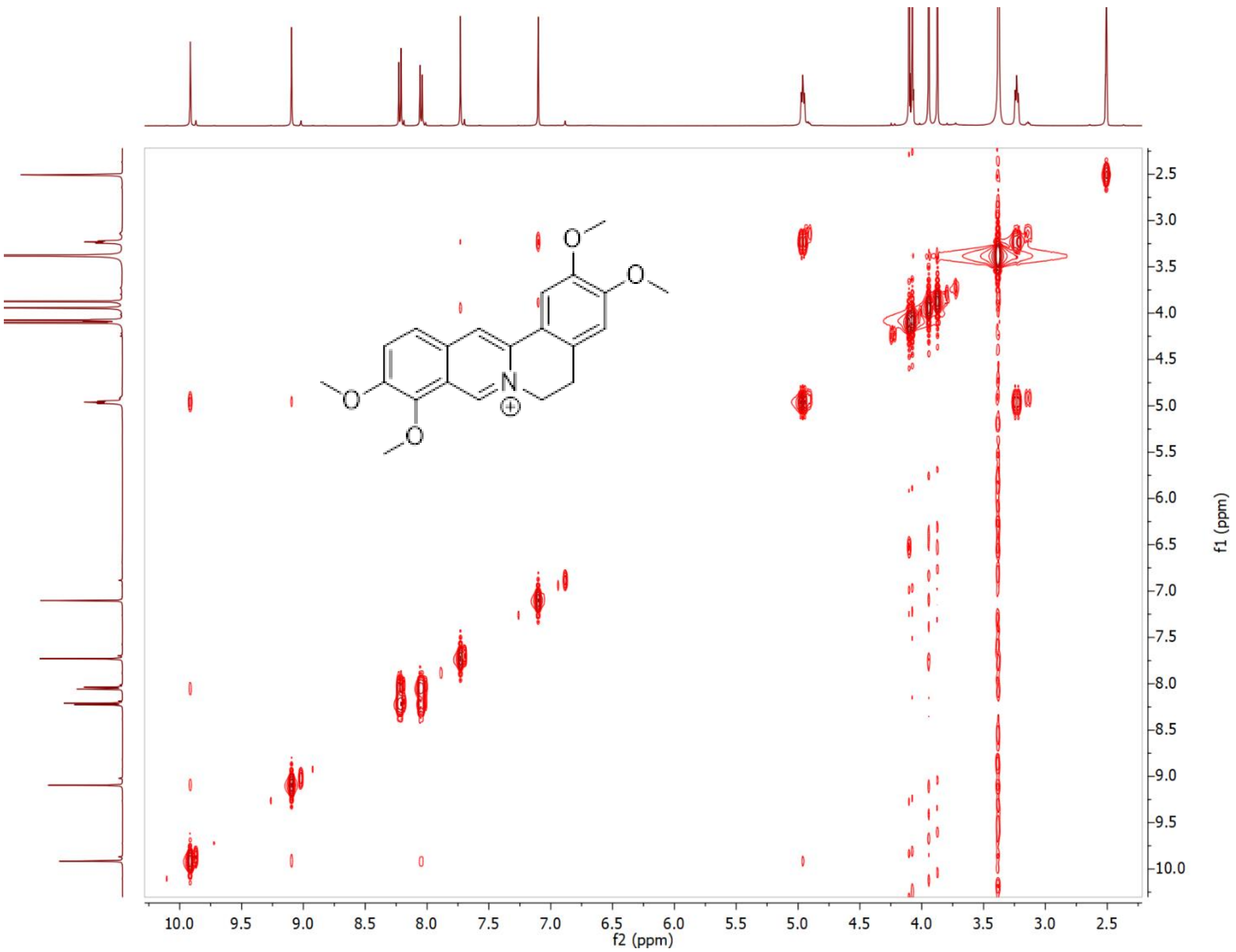

Figure S6. ^{1}H-NMR spectrum of compound **2** (DMSO-d6)

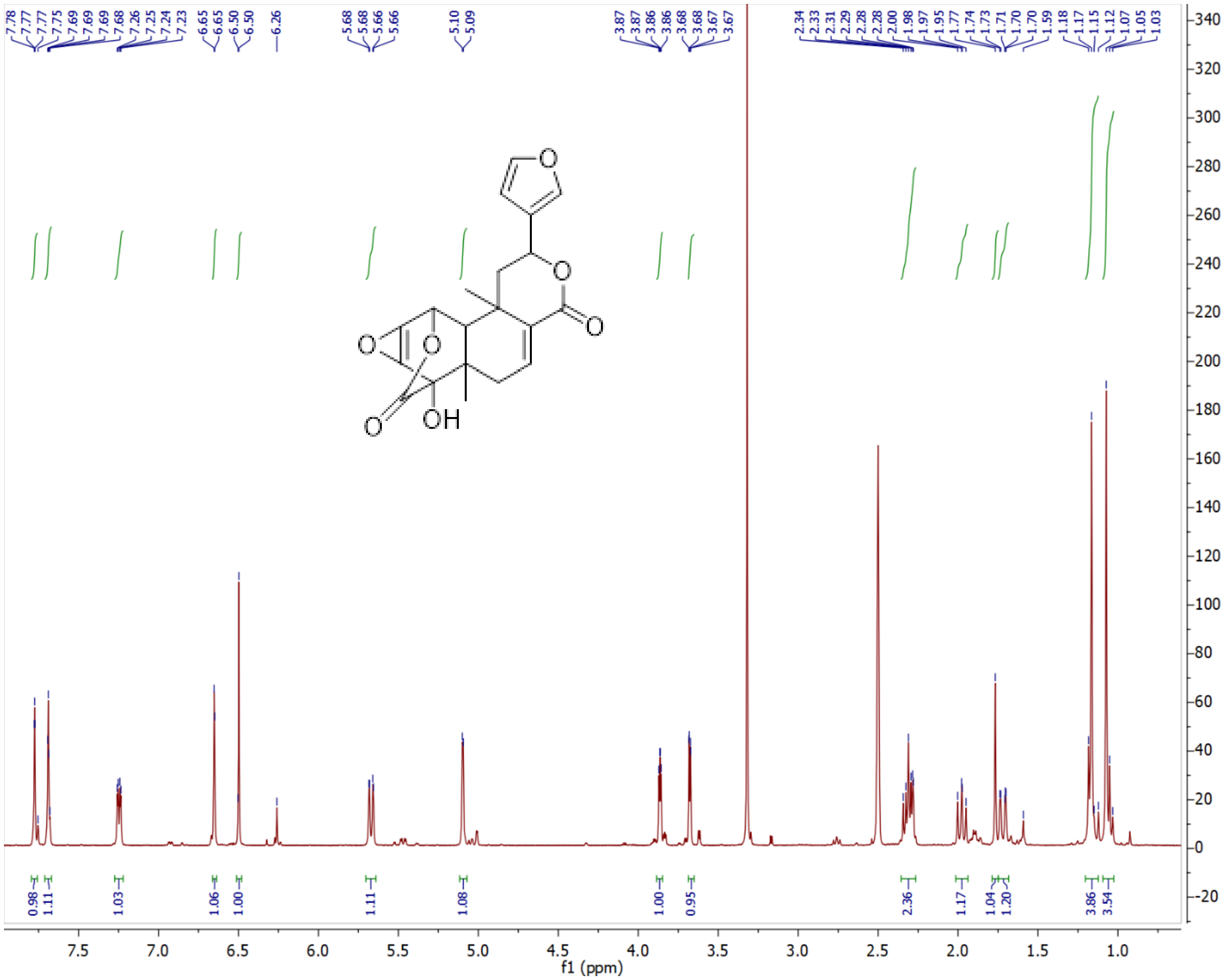

Figure S7. ^{13}C-NMR spectrum of compound **2** (DMSO-d6)

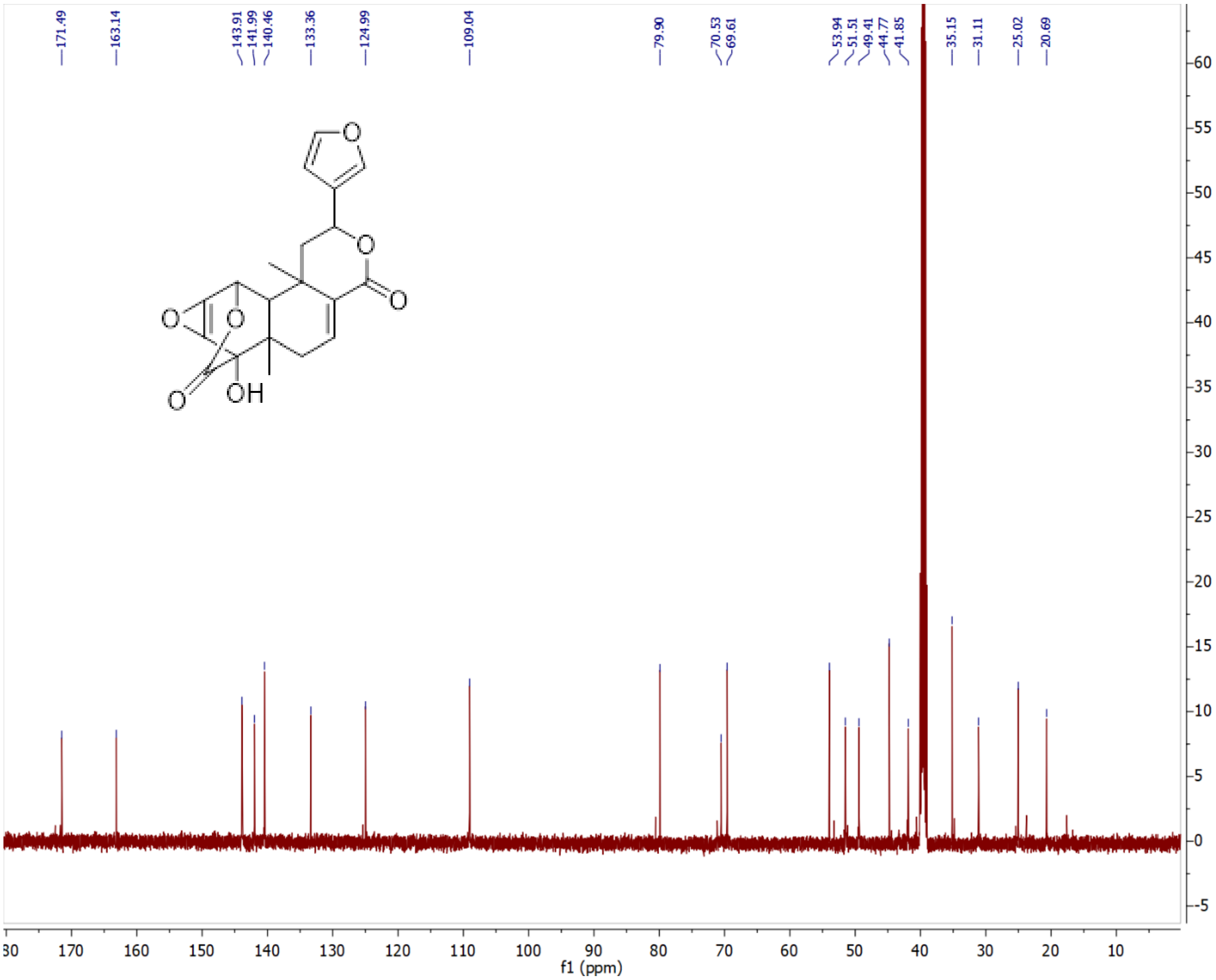

Figure S8. HSQC spectrum of compound **2** (DMSO-d6)

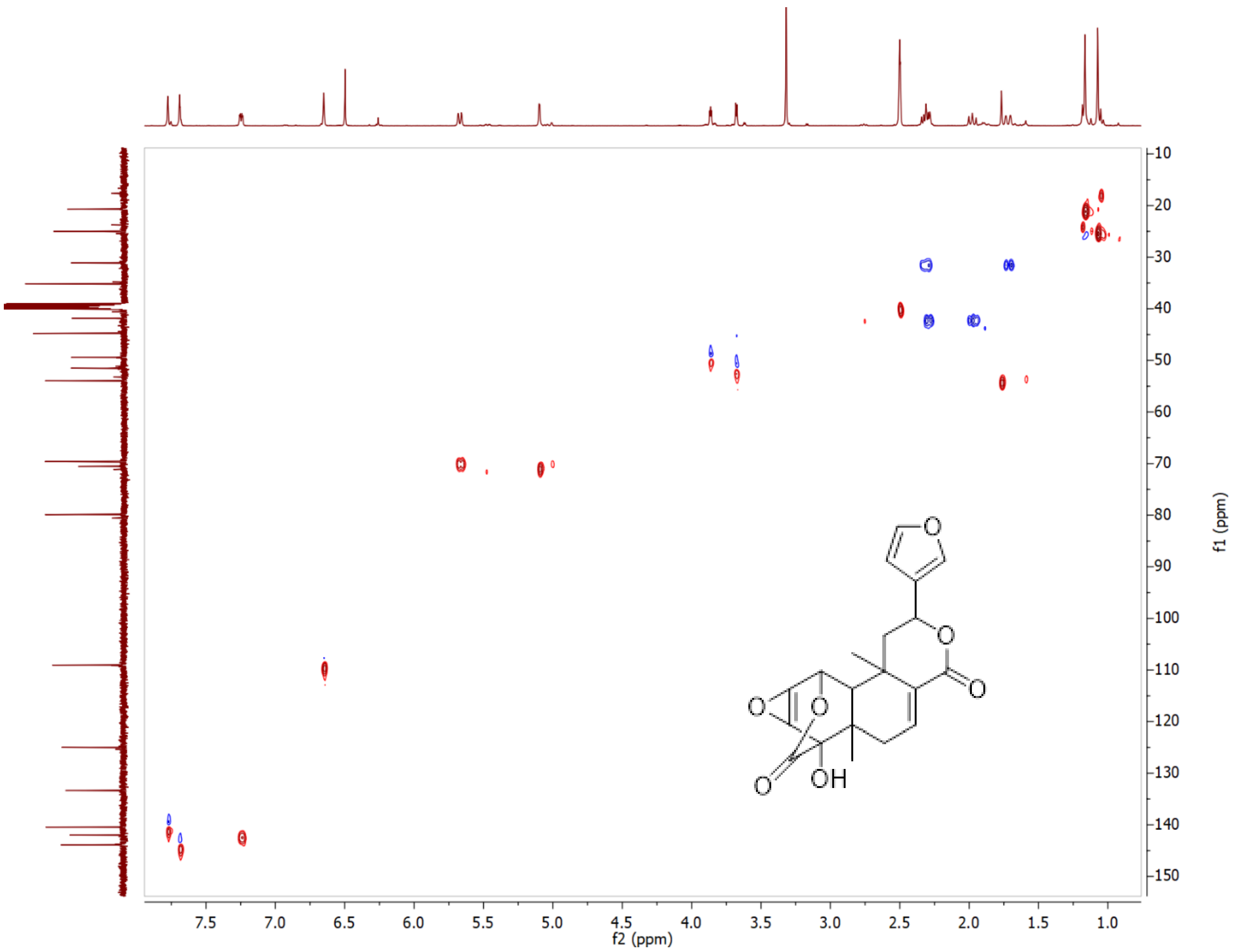

Figure S9. HMBC spectrum of compound **2** (DMSO-d6)

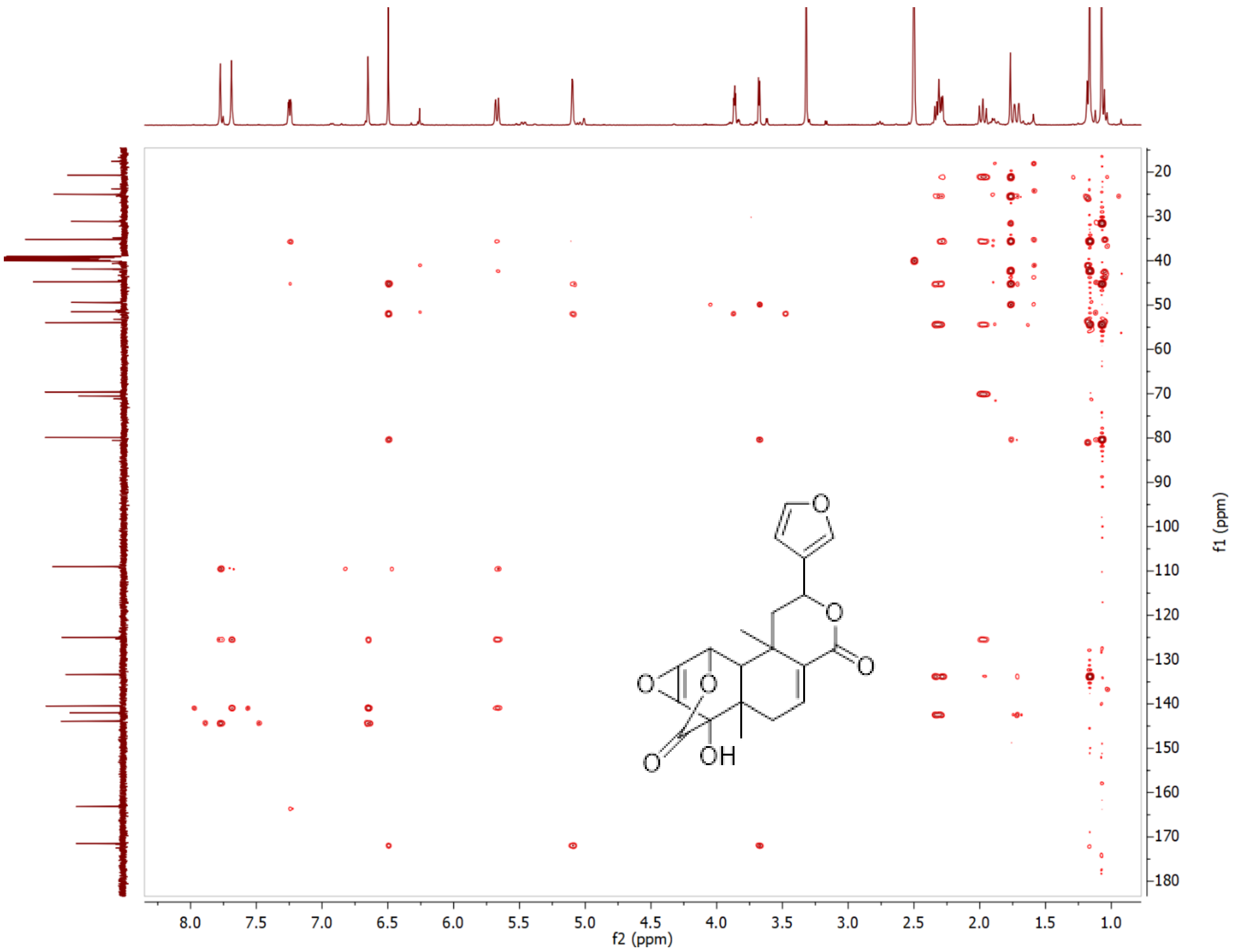

Figure S10. COSY spectrum of compound **2** (DMSO-d6)

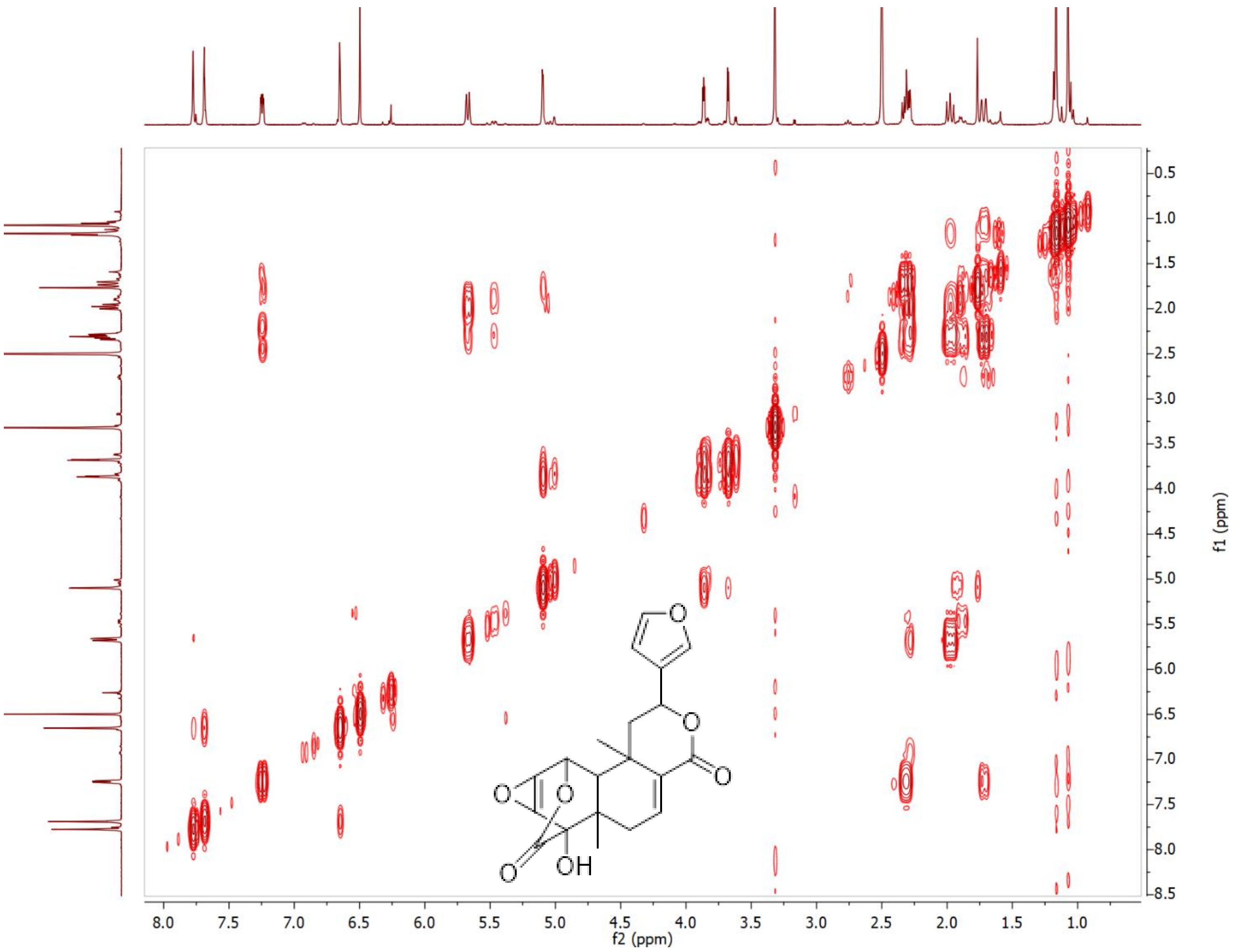

Supplement: Supplementary file 1 [file molecules-29-02373-s001.zip › molecules-3002506-supplementary.pdf]
